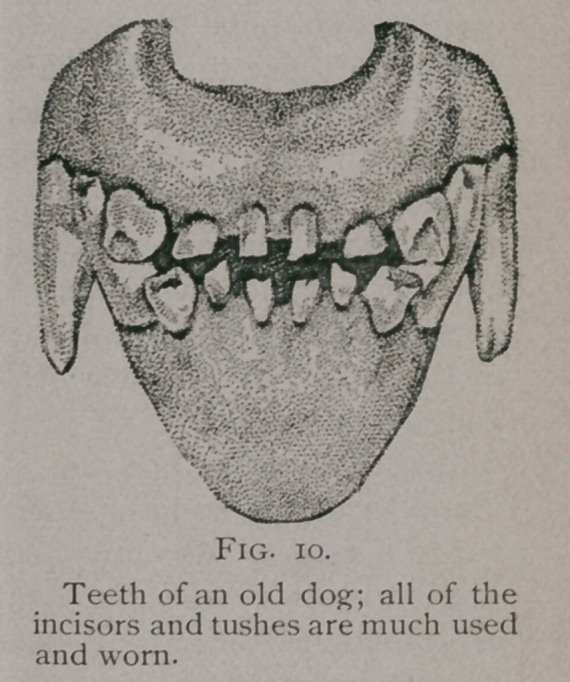# Age of the Dog

**Published:** 1891-12

**Authors:** R. S. Huidekoper

**Affiliations:** Vet.


### AGE OF THE DOG.

### FROM SAME.

---

The age of the dog is a matter of considerable importance. While the well-bred hunting-dogs, hounds, setters, and pointers, and those of other races which are kept for breeding purposes, have almost invariably a verified and registered pedigree, which is stereotyped on the end of the owner's tongue, with the date of the animal's birth, yet there are large a number of dogs, especially of the races which are kept for house-dogs and pets, which, from their appearance, are of undoubted good breeding, but which, from change of owners by sale, theft, or in the many other ways peculiar to dogs, lose their records In view of the great prices which these animals sometimes bring, it becomes needful to at least approximately determine their age and to estimate the number of years which they still have to live, and will be of use to their owners. The smaller races of dogs usually live to a greater age than the larger. The Mastiff, St. Bernard, and Great Dane rarely exceed the age of ten years ; the hound, setter, and pointer may live two or three years longer, and the terriers have been known to live to seventeen, nineteen and twenty-two years of age.

### Dentition.

Formula

- Temporary, . . . $\frac{3 \cdot 1 \cdot 3}{3 \cdot 1 \cdot 4} = 30$

- Permanent, . . $\frac{3 \cdot 1 \cdot 3 \cdot 3}{3 \cdot 1 \cdot 4 \cdot 3} = 42$

The dog has forty-two teeth,—twenty in the upper jaw and twenty-two in the lower. They are divided like those of the other animals into incisors, tusks, and molars, the latter again subdivided into premolars and post-molars.

### Incisors.

There are six incisors in each jaw, known as the *pinchers*, *intermediate*, and *corner* teeth. Those of the upper jaw are larger than those of the lower; the corner teeth are the largest, the intermediate are next in size, and the pinchers are the smallest. The incisor teeth have a large crown, on the extremity of which there are three eminences or tubercles, that in the centre being the largest, which gives it somewhat the form of a clover leaf, or *fleur de lis*. The internal face is beveled off and separated from the root of the tooth by a distinct ridge. The external face is convex in both directions, and is smooth and shiny. The root is very large, it is flattened from side to side, and is separated from the crown by a well-developed neck. The root is very firmly imbedded in the deep alveolar cavities. The virgin root contains a large dental pulp-cavity, which, however, becomes obliterated at an early period. When the incisors are worn by the friction of food and the thousand-and-one foreign bodies, which the dog "handles" with its mouth, the central tubercle is first used, then the lateral tubercles, and last the whole crown is worn, showing an irregular table of dentine, surrounded by enamel, and having in its centre a dark spot corresponding to the obliterated dental cavity.

The surface of the teeth is of a brilliant white in the dog, they are not covered with cement as in the other animals. The temporary and permanent incisors are alike in form and shape, but the former are very much smaller than the latter, and, as the head and jaw of the young dog are relatively large, the temporary incisors cannot occupy the whole arch, and so have spaces between them showing the gum, while the larger, permanent teeth when virgin are just in contact with each other by their free extremities.

### Tusks.

The dog has four tusks which are very prominent, and give the name of *canine teeth* to the corresponding teeth which resemble them in the other animals. They are placed one each side of each jaw, dividing the space between the incisive arch and the arches of the molars, unevenly; the inferior tusks are nearer to the incisive arch, while the superior ones are nearer the molars, and when the jaws are closed the tusks cross each other, coming in contact by the postero-external surface of the inferior tusk and the antero-internal face of the superior tusk. The temporary tusks are smaller, longer, more curved, and more pointed than the permanent ones.

### Molars.

Fig. 1.

Permanent dentition of the dog.

There are six molars in each arch of the upper jaw and seven in each arch of the lower. They increase in size from the first premolar to the fourth of the upper jaw, and to the first post-molar (fifth of the lower jaw), and then diminish in size at the next to last and last molar. They have large, bulbous crowns with pointed eminences, which serve for the tearing rather than the grinding of food. In the upper jaw the first three premolars are unilobular, the last is bilobular, and the last two post-molars have flat crowns. In the lower jaw the four premolars are unilobular, the first molar has three eminences, and the last two have two.

When the teeth of the dog have accomplished their eruption, they cease to grow and their roots remain firmly fixed in the alveolar cavities.

In the first dentition only the premolars are found; the post-molars appear as permanent teeth.

The molars in the dog do not form simple arches as in the other animals, but each arch represents a double curve; so that the two arches on either jaw make the form of a lyre, with its base backward and its apex forward, the widest portion of which is on a line between the last premolar and the first molar. The form or curve of the arches varies considerably with the different races of dogs, and increases greatly in those breeds which, by domestication, have had their heads and maxillæ shortened. These changes may almost approach the nature of a deformity, and the last premolar and first molar may be crowded entirely out of the line of the other teeth, or may be absent, as is sometimes seen in the pug or fancy bull-dogs.

FIG. 2.
Skull of a greyhound, under surface.

FIG. 3.
Skull of a pug, under surface.

Dr. James A. Waugh informs me that in the Mexican hairless dogs the teeth are generally irregular in number and relative position in the jaws. They have no tubercles or trefoils on the table surface of the incisor teeth. They have no canine teeth, or tushes, in either jaw. He has met with some half-breeds that had canine teeth in the upper or the lower jaw, but found no cases with those teeth in both jaws. The molars vary in number and relative position in different animals. The development of the adult dentition is much slower and later than in other classes of dogs.

One Mexican hairless dog, aged 2 years, had five superior incisors, no canine teeth, two superior molars on each side, one large molar and one very small molar behind the large one; two inferior incisors, no canine teeth, two inferior molars on each side, one large molar and one very small molar behind the large one. Total number of teeth, 15.

A Mexican hairless bitch, aged 2 years, had four superior incisors in front and one on right side midway between the front teeth and the molars, no canine teeth, two superior molars on each side, one small molar and one large molar behind the smaller one; seven inferior incisors in front, and two on each side midway between the front teeth and the molars, no canine teeth, two inferior molars on each side, one large molar and one small molar behind the large one. Total number of teeth, 24.

### Determination of Age by the Teeth.

From the evidences furnished by the teeth the age is divided into three periods :—

1. Eruption of the temporary teeth.
2. Eruption of the permanent teeth.
3. Wearing of the permanent teeth.

### Eruption of the Temporary Teeth.

*At Birth.*—Puppies may be born with all of their temporary teeth, but if the teeth have not appeared the eruption commences at once by incisors and tusks of the upper jaw, and the entire dentition is effected within the first three weeks at longest. (Fig. 4.)

*Two to Four Months.*—A month before the eruption of the permanent teeth the temporary pinchers and often the intermediate teeth in both jaws become loose and fall out; at this time the points of the permanent teeth can be felt by pressing on the gums. (Fig. 5.)

### Eruption of the Permanent Teeth.

The permanent teeth replace those of first dentition several months earlier in the large races of dog than in the terrier varieties. Medium-sized dogs, setters, etc., make the change later than the large dogs and earlier than the terriers.

*Five to Eight Months.*—According to the race of dog, the permanent teeth appear rapidly in the following order: pinchers, intermediate, corners, and tusks. The incisors do not come out obliquely, as in the herbivora. It is worthy of note that, while the herbivora do not have all of their permanent dentition until they have arrived at their full development of body, the carnivora have theirs long before they have attained their growth and before the consolidation of their bones.

FIG. 4.

FIG. 5.

FIG. 6.

FIG. 4.—Teeth of puppy two months old.

FIG. 5.—Teeth of dog four months old; the incisors are loose and ready to fall out.

FIG. 6.—Teeth of dog one year old, showing the incisors and tushes fresh and unworn.

*One Year.*—For the first few months after their appearance there is no alteration in the teeth; at one year they are pure white and the tubercles on the incisors are intact. (Fig. 6.)

*Fifteen Months.* From fifteen months on, commences the wearing away of the teeth. This is first seen on the inferior pinchers, while the tusks and other teeth remain fresh and white in color.

*Eighteen Months to Two Years.*—At about this time the tubercles of the inferior pinchers become worn down and those of the other incisors commence to be used. (Fig. 7.)

*Two and One-half Years to Three Years.*—After two and a half years the tubercles of the inferior intermediate teeth are worn away, the incisors of the upper jaw shows signs of use, and all of the teeth commence to lose their fresh, white appearance and become yellowish and discolored. (Fig. 8.)

*Three and One-half Years to Four Years.*—The pinchers of the upper jaw become worn down and are followed by the wearing of the superior intermediate teeth.

The discoloration of the teeth increases and the tusks become yellow and dirty in appearance.

After this the leveled and broken teeth give no indication of the age. A Great Dane, which for twelve years had only been fed at the table on tid-bits, and a poodle of thirteen years, had teeth as fresh as those of an ordinary dog at four or five; and bull-terriers and others at four or five may have the incisors nearly worn away.

FIG. 7.

Teeth of dog eighteenth months old, showing use of the inferior pinchers. The tushes are still fresh and white.

FIG. 8.

Teeth of dog two and a half years old, showing use of inferior pinchers and intermediate teeth.

The leveling of the teeth of the dog consists of the wearing away of the middle tubercle to the level of the lateral tubercles and the wearing of the enamel from these. As we have just seen, the leveling commences with the inferior pinchers, is followed by that of the inferior intermediate teeth, and does not commence on the superior incisors until it is complete on the inferior ones; with the superior incisors it commences with the pinchers and follows with the intermediate teeth; the tusks do not commence to wear until the use of the incisors is marked. The leveling of the teeth may, however, take place much more rapidly or more slowly than has been indicated above, and may even be very irregular in the teeth which it first effects, according to the nature of the food upon which the animal is nourished.

Dogs fed on meat and those which have bones to knaw use their teeth more rapidly than those which are fed on soups, broken bread, and vegetables. Dogs which are pugnacious, and those which are taught to swing on ropes or straps and to fetch objects, such as sticks, stones, etc., use their teeth rapidly and are apt to have their tusks much worn. These dogs also frequently have irregular mouths, from broken teeth. From these causes it sometimes happens, especially with the larger dogs, that the teeth are so altered after three years that no intelligible deduction can be drawn from the appearance of the mouth.

FIG. 9.

Teeth of dog over three years old, showing use of all of the incisors; the tushes are becoming yellow.

FIG. 10.

Teeth of an old dog; all of the incisors and tushes are much used and worn.

### DETERMINATION OF AGE FROM OTHER SIGNS.

The young dog is born with its eyelids closed; these do not open until the ninth or tenth and sometimes as late as the fifteenth day.

Young dogs have heads which are large in proportion to the size of the face.

Old dogs become gray around the nose, eyes, and forehead; their noses become larger, and the skin over the whole face becomes wrinkled. The lips become everted and show the red mucous membrane along their irregular borders. The skin, at the ordinary points of pressure in decubitus, especially in large dogs, become denuded of hair and develops an epithelial growth which is almost horny in character; so that in old dogs we find callous point at the elbow, hock, salient points of the pelvis, and sometimes on the shoulder.

## Figures and Tables

**Fig. 1. f1:**
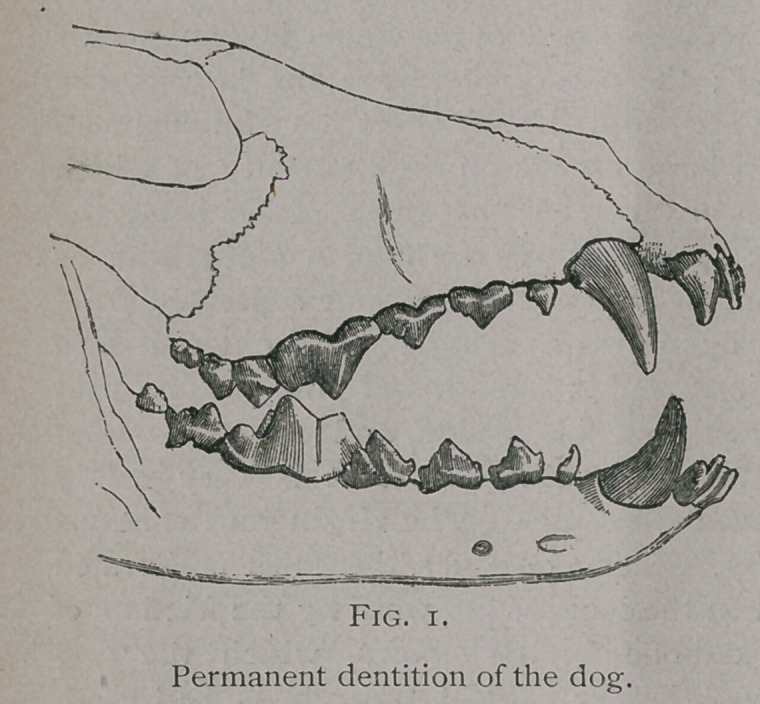


**Fig. 2. f2:**
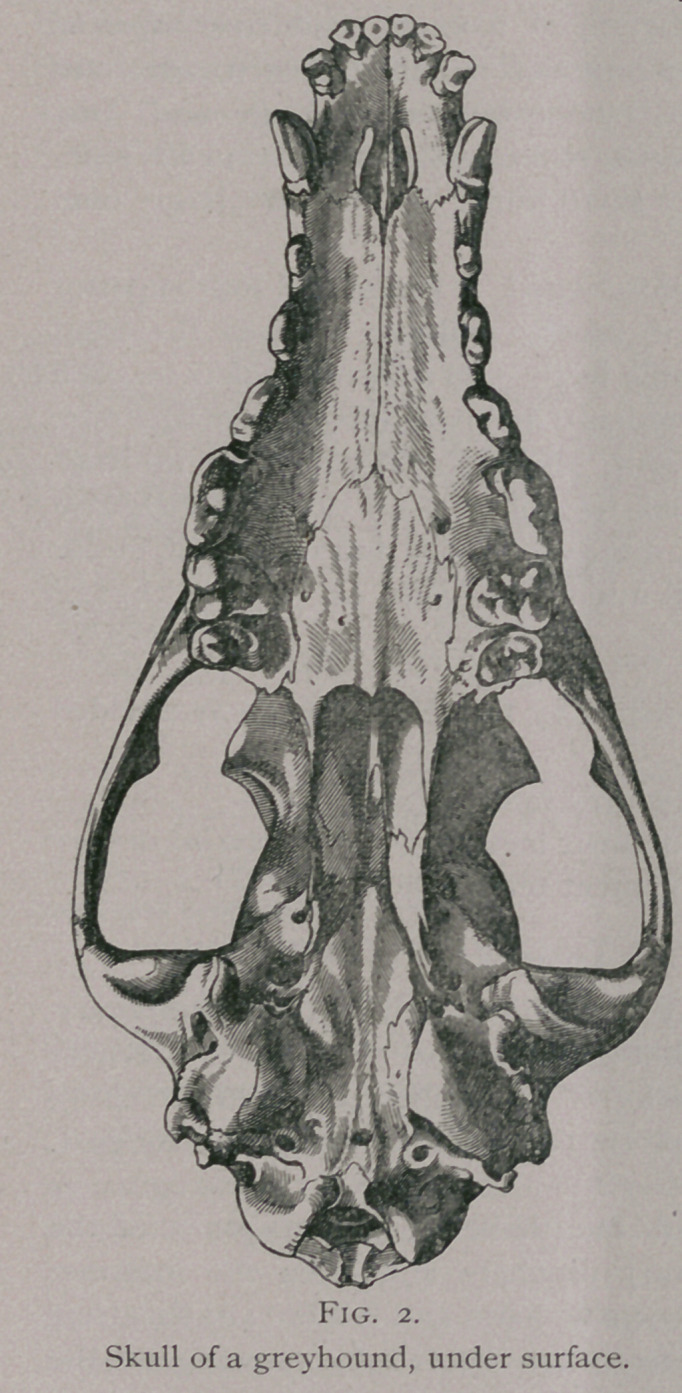


**Fig. 3. f3:**
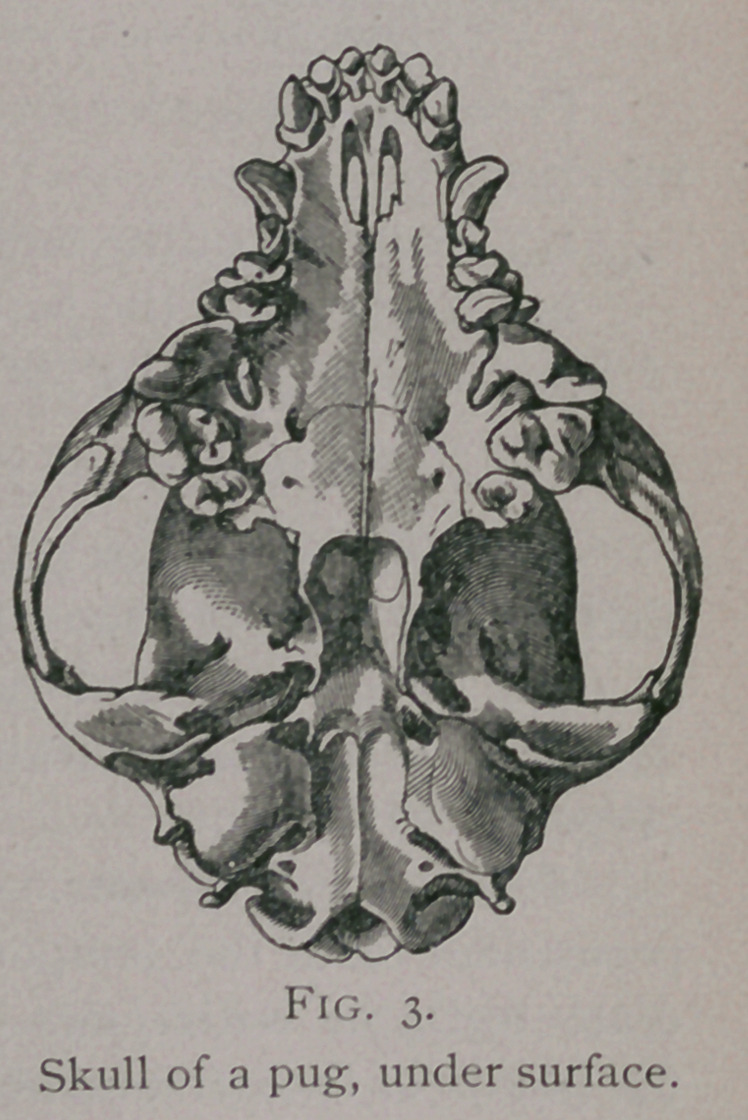


**Fig. 4. Fig. 5. Fig. 6. f4:**
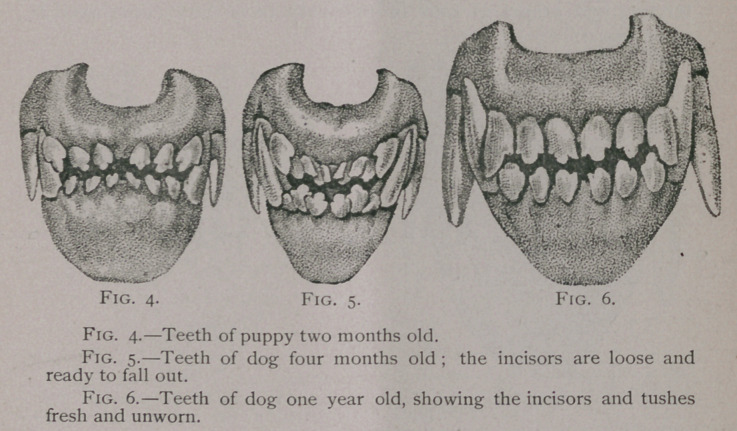


**Fig. 7. f5:**
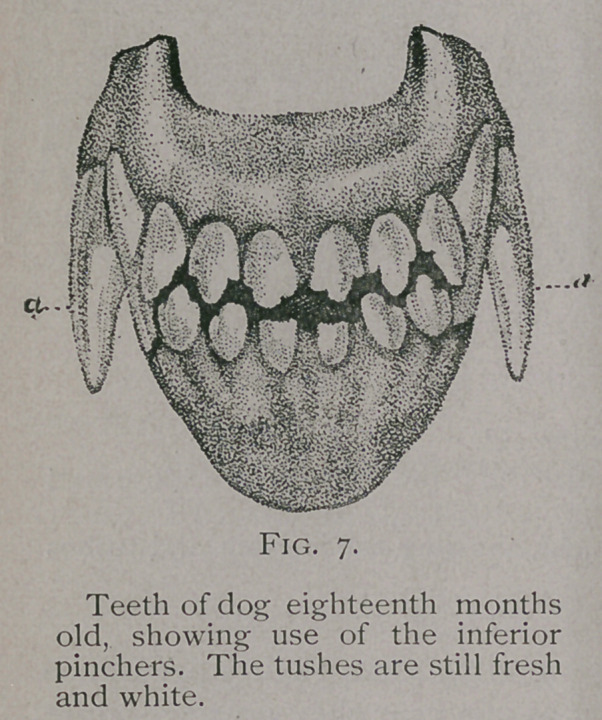


**Fig. 8. f6:**
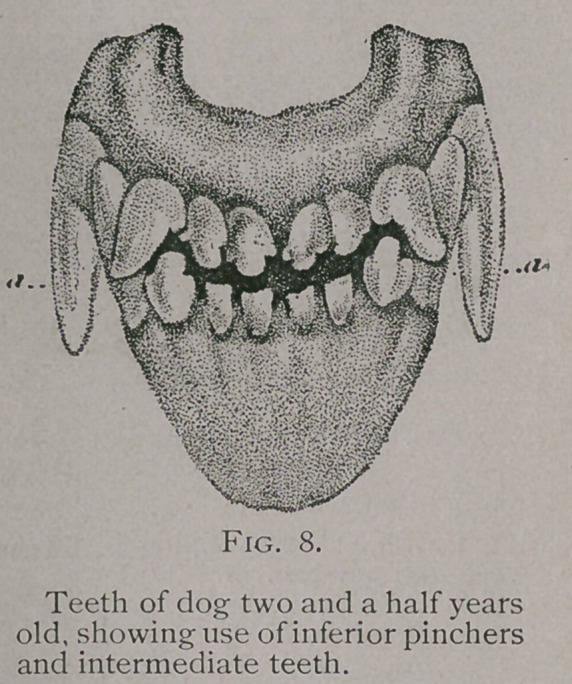


**Fig. 9. f7:**
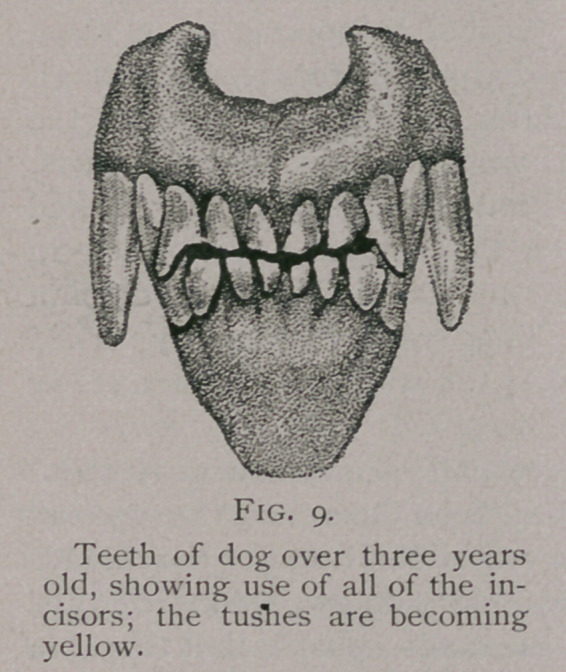


**Fig. 10. f8:**